# Does fertilization with dehydrated sewage sludge affect *Terminalia argentea* (Combretaceae) and associated arthropods community in a degraded area?

**DOI:** 10.1038/s41598-020-68747-z

**Published:** 2020-07-16

**Authors:** Jó Cássio Nascimento Carvalho, Farley William Souza Silva, Germano Leão Demolin Leite, Alcinei Mistico Azevedo, Gustavo Leal Teixeira, Marcus Alvarenga Soares, José Cola Zanuncio, Jesusa Crisostomo Legaspi

**Affiliations:** 1 Insetário G.W.G. Moraes, Instituto de Ciências Agrárias, Universidade Federal de Minas Gerais, 39.404-547 Montes Claros, Minas Gerais Brasil; 2 Centro de Ciências Biológicas e da Natureza, Universidade Federal do Acre, 69920-900, Rio Branco, Acre, Brasil; 3 Programa de Pós-Graduação em Produção Vegetal, Universidade Federal dos Vales do Jequitinhonha e Mucuri, 39100-000 Diamantina, Minas Gerais Brasil; 4 Departamento de Entomologia/BIOAGRO, Universidade Federal de Viçosa, 36570-900 Viçosa, Minas Gerais Brasil; 5 Center for Medical, Agricultural and Veterinary Entomology, United States Department of Agriculture, Tallahassee, FL 32308 USA

**Keywords:** Ecology, Environmental sciences, Entomology

## Abstract

Nutrients from dehydrated sewage sludge play an essential role in the development of many plants such as *Terminalia argentea*, in the recovery of degraded areas. The aims were to assess the abundance, diversity and species richness of phytophagous, pollinators and predators arthropods, as well as the percentage of defoliation of *T. argentea* trees, fertilized (or not) with dehydrated sewage sludge in a degraded area. The abundance, diversity and species richness of phytophagous Coleoptera and total predators (predator insects + protocooperating ants + spiders); abundance and species richness of Diptera, pollinator insects, spiders, and predators (predator insects + spiders) were higher on trees fertilized with dehydrated sewage sludge. The abundance of phytophagous Coleoptera declined with the presence of phytophagous Hemiptera and protocooperating ants; population of phytophagous Orthoptera declined in response to phytophagous Coleoptera and total predators; the numbers of the leafminer *Lyriomyza* sp. directly increased with the numbers of spiders. The ecological indices of phytophagous, pollinators, and predator arthopods increased on *Terminalia argentea* trees fertilized with dehydrated sewage sludge; such a better ecological indices in fertilized than in unfertilized trees, show it more suitable for the recovery of degraded areas. We discuss the competition between phytophagous insects groups as well as herbivory reduction by predators.

## Introduction

Sewage sludge, a residual and semi-solid material, produced as a by-product during domestic and industrial waste water treatment, is rich in organic matter, shows potential for fertilization and production of seedling substrates^1,2^. Sewage sludge can be used safely in agriculture and forests plantations as fertilizer and in the recovery of degraded areas, with a low-cost alternative to reduce the environmental impacts and to avoid contamination of the human food chain^3–5^. Furthermore, dehydrated sewage sludge (DSS) does not affect the heavy metal contents in grains of maize, *Zea mays* L. (Poales: Poaceae) and cowpea, *Vigna unguiculata* (L.) Walp. (Fabales: Fabaceae)^6^.

*Terminalia argentea* Mart. & Zucc (Combretaceae), a secondary native tree from the Southeastern and Central-western Brazil, is used for landscaping, wood and coal production, civil construction and the recovery of degraded areas^7^. Continuous release of exudates by *T. argentea* in the trunk is typical due to pathogens attack, affecting the constant visitation by *Trigona branneri* (Crockere) and *Mesembrinella bicolor* (Fabricius) (Hymenoptera: Apidae)^8^.

Insect diversity may be used to assess the recovery of degraded area, as these organisms easily respond to environmental changes^9^. Different orders of insects, with a large number of families and species, including Coleoptera, are widely used as a bioindicator^5,10^. Nutritional indices and chemical plant defenses are associated with factors such as fertilization and plant development (i.e. age), affecting phytophagous insects and therefore, the natural enemies’ diversity^5,11–13^. Sewage sludge increases the humus content in the soil and it is rich in macro (e.g. N, P and K) and micronutrients (e.g. Cu and Zn)^14^, favoring plants and, consequently, insect development.

The aims here were to assess for 24 months the ecological indices (abundance, diversity and species richness) and ecological processes (herbivory and predation) of phytophagous, pollinators and predators arthropods on *T. argentea* trees, fertilized (or not) with DSS in a degraded area. We hypothesize that (i) *T. argentea* trees resemble living islands, and that the fertilization with DSS may increase the canopy size (canopy islands), and thus accommodate larger numbers of phytophagous, pollinators and predators arthropods (> the equilibrium theory of island biogeography—ETIB)^5,15–17^; (ii) there is competition between groups of phytophagous insects, such as hemipterans, coleopterans and orthopterans^18,19^; and (iii) arthropod predators, such as insects and spiders, reduce the number of phytophagous insects and thus herbivory on *T. argentea* trees^19–21^.

## Results

### *Terminalia argentea* trees and arthropods

**Figure 1:**
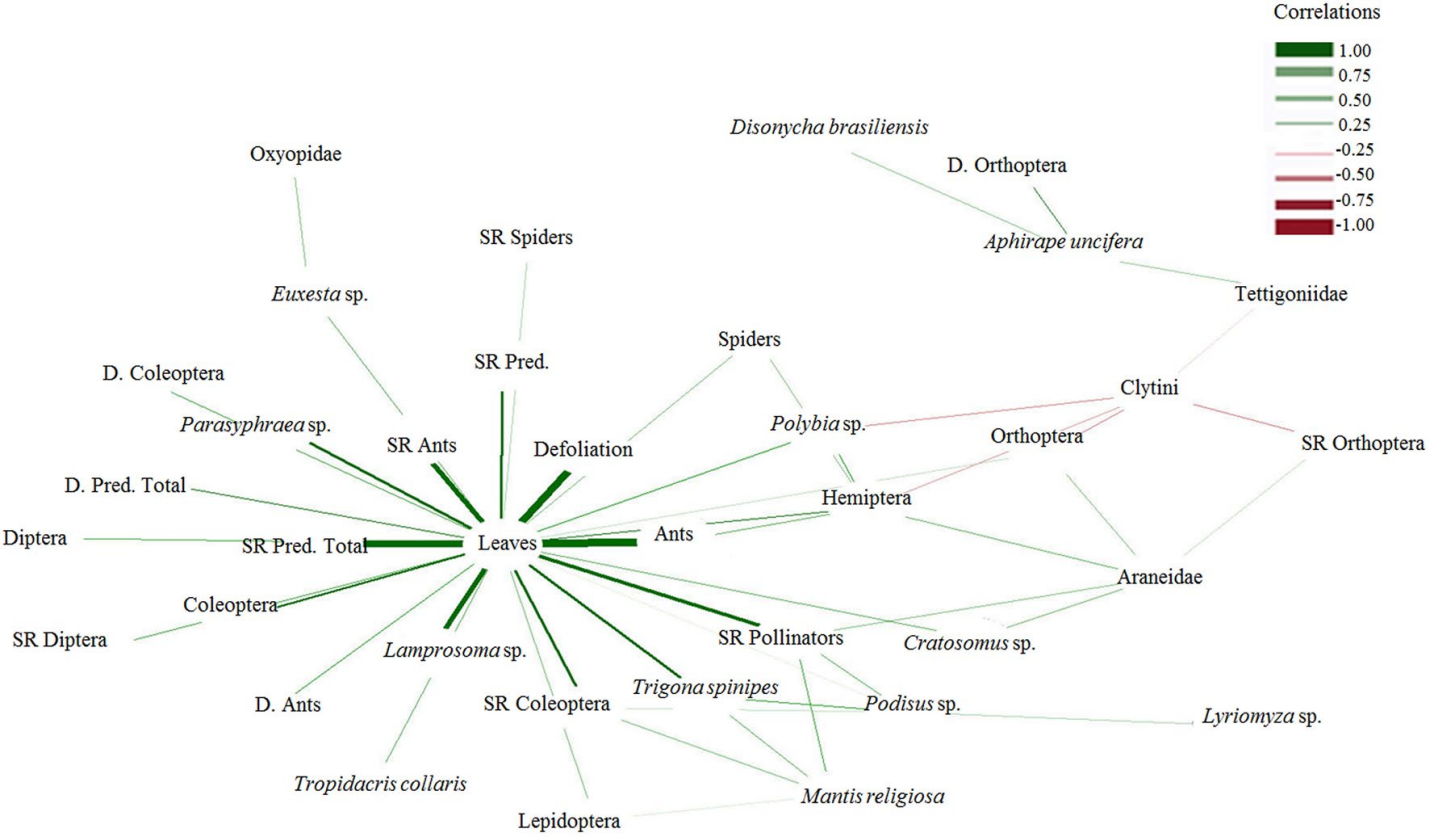
Estimated network structures based on the Spearman correlation (P < 0.05) generated for total leaves per tree, defoliation (%), and the abundances of *Aphirape uncifera*, Araneidae, spiders, phytophagous Coleoptera, Clytini, *Cratosomus* sp., Diptera, *Disonycha brasiliensis*, *Euxesta* sp., protocooperating ants, phytophagous Hemiptera, *Lamprosoma* sp., Lepidoptera, *Mantis religiosa*, *Lyriomyza* sp. mines, phytophagous Orthoptera, Oxyopidae, *Parasyphraea* sp., *Podisus* sp., *Polybia* sp., Tettigoniidae, *Trigona spinipes*, and *Tropidacris collaris*; the diversity (D.) of protocooperating ants, phytophagous Coleoptera, total predators (predators + spiders + protocooperating ants), and phytophagous Orthoptera; and species richness (SR) of spiders, phytophagous Coleoptera, Diptera, protocooperating ants, Orthoptera, pollinators, predators, and total predadors on *Terminalia argentea* trees. n = 48.

The phytophagous Coleoptera and the abundance, diversity and species richness of total predators and Diptera, pollinators, spiders, predator abundance and species richness were higher (*P* < 0.05) on *T. argentea* trees fertilized with DSS (Table 1). Percentage of defoliation and phytophagous Coleoptera *Psiloptera* sp. (Buprestidae), Cerambycidae, *Cerotoma* sp., *Lamprosoma* sp., *Parasyphraea* sp. (Chrysomelidae) and *Cratosomus* sp. (Curculionidae); *Euxesta* sp. (Diptera: Otitidae), Lepidoptera caterpillars and *Tropidacris collaris* Stoll (Orthoptera: Romaleidae); pollinators *Trigona spinipes* Fabricius (Hymenoptera: Apidae); and predators Araneidae and Salticidae (Araneae), *Podisus* sp. (Hemiptera: Pentatomidae), *Polybia* sp. (Hymenoptera: Vespidae), protocooperating ants (Hymenoptera: Formicidae) and *Mantis religiosa* L. (Mantodea: Mantidae) were higher (*P* < 0.05) on *T. argentea* trees fertilized with DSS (Tables 2 and 3). The abundance of Coleoptera, Diptera, Hemiptera and Orthoptera, spiders and protocooperating ants; the diversity of Coleoptera, protocooperating ants and total predator; the species richness of Coleoptera, Diptera, pollinators, protocooperating ants, spiders, predators and total predator; the percentage of defoliation; the numbers of phytophagous insects *Cratosomus* sp., *Euxesta* sp., *Lamprosoma* sp., Lepidoptera, *Parasyphraea* sp. e *T. collaris*, pollinators *T. spinipes*, and predators Pentatomidae and *Polybia* sp. increased with the total numbers of *T. argentea* leaves (Fig. 1).

**Table 1:** The abundance (Abun.), diversity (D), and species richness (RS) of phytophagous insects, pollinators, spiders, predators (predators + spiders) (Pred.), total predators (predators + spiders + protocooperating ants) (Tot. Pred.) on *Terminalia argentea* Mart & Zucc (Combretaceae) trees (mean ± SE) fertilized or non-fertilized with dehydrated sewage sludge in degraded area.

Ecological indices	Sewage sludge		Wilcoxon test	
	Fertilized	Non-fertilized	VT*	*P*
Abund. Coleoptera	9.79 ± 1.32	2.54 ± 0.79	4.3	0.00
D Coleoptera	7.63 ± 1.08	2.68 ± 0.50	3.1	0.00
SR Coleoptera	4.21 ± 0.35	1.54 ± 0.28	4.5	0.00
Abund. Diptera	1.21 ± 0.55	0.00 ± 0.00	3.3	0.00
D Diptera	0.00 ± 0.00	0.00 ± 0.00	–-	–-
SR Diptera	0.38 ± 0.10	0.00 ± 0.00	3.3	0.00
Abund. Orthoptera	1.00 ± 0.28	0.38 ± 0.11	1.6	0.06
D Orthoptera	0.30 ± 0.12	0.30 ± 0.12	–-	–-
SR Orthoptera	0.50 ± 0.10	0.33 ± 0.09	1.2	0.12
Abund. pollinators	10.33 ± 3.01	0.38 ± 0.26	4.3	0.00
D pollinators	0.00 ± 0.00	0.00 ± 0.00	–-	–-
SR pollinators	0.83 ± 0.13	0.08 ± 0.05	4.4	0.00
Abund. spiders	4.38 ± 1.38	1.50 ± 0.36	2.3	0.01
D spiders	2.25 ± 0.65	2.35 ± 0.71	0.0	0.48
SR spiders	1.75 ± 0.21	1.25 ± 0.27	1.8	0.04
Abund. Pred	6.21 ± 1.46	1.75 ± 0.40	4.1	0.00
D Pred	4.42 ± 0.94	3.06 ± 0.81	0.9	0.18
SR Pred	3.04 ± 0.22	1.42 ± 0.31	3.8	0.00
Abund. Tot. Pred	37.83 ± 3.99	7.17 ± 1.05	5.6	0.00
D Tot. Pred	14.18 ± 1.36	8.89 ± 1.30	2.8	0.00
SR Tot. Pred	8.33 ± 0.40	3.83 ± 0.43	5.2	0.00

n = 24 per treatment. VT* = value of the test. –- = it was not possible to generate due to zero in the treatments.

**Table 2:** The abundance of phytophagous insects on *Terminalia argentea* Mart & Zucc (Combretaceae) and defoliation (%) of trees (mean ± SE) fertilized or non-fertilized with dehydrated sewage sludge in a degraded area.

Order: family	Species	Sewage sludge		Wilcoxon test	
		Fertilized	Non-fertilized	VT*	*P*
Coleoptera					
Buprestidae	*Psiloptera* sp.	0.21 ± 0.13	0.00 ± 0.00	1.8	0.04
Cerambycidae	Non-identified	0.25 ± 0.10	0.00 ± 0.00	2.3	0.01
Chrysomelidae	*Alagoasa* sp.	0.00 ± 0.00	0.42 ± 0.41	1.0	0.16
	Clytrini	0.25 ± 0.09	0.54 ± 0.14	1.4	0.08
	*Cerotoma* sp.	0.46 ± 0.19	0.08 ± 0.05	1.6	0.05
	*Diabrotica speciose* Germar	0.08 ± 0.05	0.08 ± 0.05	0.0	0.50
	*Disonycha brasiliensis* Costa Lima	0.13 ± 0.06	0.08 ± 0.05	0.5	0.32
	*Eumolpus* sp.	0.04 ± 0.04	0.00 ± 0.00	1.0	0.16
	*Gynandrobrotica* sp.	0.04 ± 0.04	0.00 ± 0.00	1.0	0.16
	*Lamprosoma* sp.	1.63 ± 0.25	0.13 ± 0.09	5.2	0.00
	*Parasyphraea* sp.	1.92 ± 0.58	0.04 ± 0.04	3.8	0.00
	*Walterianella* sp.	0.00 ± 0.00	0.13 ± 0.06	1.8	0.04
	*Wanderbiltiana* sp.	0.08 ± 0.05	0.04 ± 0.04	0.6	0.28
Curculionidae	Non-identified	0.04 ± 0.04	0.00 ± 0.00	1.0	0.16
	*Cratosomus* sp.	1.13 ± 0.42	0.00 ± 0.00	3.1	0.00
	*Diorymerus* sp.	1.17 ± 0.48	0.38 ± 0.29	1.5	0.07
	*Lordops* sp.	0.00 ± 0.00	0.04 ± 0.04	1.0	0.16
Tenebrionidae	*Epitragus* sp.	0.29 ± 0.12	0.04 ± 0.04	1.8	0.04
Diptera					
Agromyzidae	*Lyriomyza* sp.	0.79 ± 0.55	0.00 ± 0.00	1.4	0.08
Otitidae	*Euxesta* sp.	0.42 ± 0.17	0.00 ± 0.00	2.8	0.00
Hemiptera	–	21.04 ± 8.46	2.79 ± 1.17	3.1	0.00
Blattodea					
Termitidae	*Nasutitermes* sp.§	4.17 ± 2.88	0.00 ± 0.00	1.4	0.08
Lepidoptera	Non-identified	0.29 ± 0.09	0.04 ± 0.04	2.3	0.01
Orthoptera					
Gryllidae	Non-identified	0.04 ± 0.04	0.00 ± 0.00	1.0	0.16
Proscopiidae	*Cephalocoema* sp.	0.17 ± 0.16	0.00 ± 0.00	1.0	0.16
Romaleidae	*Tropidacris collaris* Stoll	2.08 ± 0.48	0.54 ± 0.19	3.1	0.00
Tettigoniidae	Non-identified	0.79 ± 0.25	0.38 ± 0.11	1.0	0.17
% defoliation	–	7.88 ± 0.28	3.70 ± 0.21	5.7	0.00

n = 24 per treatment. VT* = value of the test. §Observed on *T. argentea* trunk.

**Table 3:** The abundance of predators, protocooperating ants, and pollinators on *Terminalia argentea* Mart & Zucc (Combretaceae) trees (mean ± SE) fertilized or non-fertilized with dehydrated sewage sludge in a degraded area.

Order: family	Species	Sewage sludge		Wilcoxon test	
		Fertilized	Non-fertilized	VT*	*P*
Araneae					
Araneidae	Non-identified	2.96 ± 1.39	0.46 ± 0.14	1.8	0.04
Anyphaenidae	*Teudis* sp.	0.00 ± 0.00	0.04 ± 0.04	1.0	0.16
Salticidae	Non-identified	0.54 ± 0.14	0.25 ± 0.12	1.8	0.04
	*Aphirape uncifera* Tullgren	0.04 ± 0.04	0.21 ± 0.10	1.4	0.08
	*Uspachus* sp.	0.13 ± 0.09	0.04 ± 0.04	0.6	0.27
Sparassidae	*Quemedice* sp.	0.04 ± 0.04	0.13 ± 0.06	1.0	0.16
Oxyopidae	Non-identified	0.42 ± 0.14	0.17 ± 0.07	1.4	0.09
	*Oxyopes salticus* Hentz	0.04 ± 0.04	0.04 ± 0.04	0.0	0.50
Tetragnathidae	*Leucauge* sp.	0.08 ± 0.05	0.04 ± 0.04	0.6	0.28
Thomisidae	*Aphantochilus rogersi* O.P.Camb	0.08 ± 0.05	0.08 ± 0.05	0.0	0.50
	*Tmarus* sp.	0.04 ± 0.04	0.04 ± 0.04	0.0	0.50
Hemiptera					
Pentatomidae	*Podisus* sp*.*	0.29 ± 0.17	0.00 ± 0.00	2.1	0.02
Hymenopera					
Apidae	*Apis mellifera* L	0.08 ± 0.05	0.21 ± 0.20	0.5	0.29
	*Tetragonisca angustula* Latreille	0.08 ± 0.05	0.00 ± 0.00	1.4	0.08
	*Trigona spinipes* Fabricius	10.17 ± 3.00	0.17 ± 0.16	4.4	0.00
Formicidae	Protocooperating	28.67 ± 3.98	5.08 ± 0.18	5.2	0.00
Vespidae	*Polybia* sp.	0.75 ± 0.16	0.21 ± 0.12	3.0	0.00
Mantodea					
Mantidae	*Mantis religiosa* L	0.38 ± 0.14	0.04 ± 0.04	2.1	0.02

n = 24 per treatment. VT* = value of the test.

### Competition between phytophagous insects

The abundance of phytophagous Hemiptera and pollinators reduced (*P* < 0.05) the abundance of phytophagous Coleoptera, phytophagous Orthoptera and *T. spinipes*; the abundance of phytophagous Hemiptera and phytophagous Orthoptera reduced Clytini (Coleoptera: Chrysomelidae); the species richness of pollinators reduced (*P* < 0.05) phytophagous Coleoptera; and the species richness of phytophagous Hemiptera reduced pollinators (Table 4, Fig. 1).

**Table 4:** Simple regression equation analysis of the variables of phytophagous Coleoptera (Ab.Col.) with phytophagous Hemiptera (Ab.Hem.), pollinator insects (Ab.Pol.), protocooperating ants (Ab.Ant.), and predators (predators + spiders) (Ab.Pred.); phytophagous Orthoptera (Ab.Orth.) with Ab.Col. and total predators (predators + spiders + protocooperating ants) (Ab.Tot.Pred..); *Euxesta* sp. (Ab.Eux.) with Ab.Ant.; *Lyriomyza* sp. (Ab.Lyr.) with Araneidae (Ab.Aranei), spiders (Ab.Spid.) with Ab.Tot.Pred.; Ab.Pol. with Ab.Ant. and Ab.Tot.Pred.; *T. spinipes* (Ab.Ts.) with Ab.Ant., Ab.Tot.Pred. and Ab.Col.; Ab.Spid. with Ab.Pol. and Ab.Ts.; Ab.Aranei with Ab.Ts.; Ab.Tot.Pred. with Ab.Col.; species richness of phytophagous Coleoptera (SR.Col.) with species richness of pollinators (SR.Pol.) and predators (SR.Pred.); SR.Pol.) with phytophagous Hemiptera (SR.Hem.); diversity of phytophagous Orthoptera (D.Orth.) with diversities of spiders (D.Spid.) and total predators (D.Tot.Pred.); protocooperating ants (D.Ant.) with phytophagous Hemiptera (D.Hem.) and D.Ara. on *Terminalia argentea* Mart & Zucc (Combretaceae) trees in a degraded area.

Equations of the simple regression	R^2^	ANOVA	
		*F*	*P*
Ab.Col. = 3.59 + 20.36 × √¯Ab.Hem. − 0.14 × Ab.Hem	0.16	4.4	0.02
Ab.Col. = 4.59 + 0.56 × Ab.Pol. − 0.01 × Ab.Pol.^2^	0.18	4.9	0.01
Ab.Col. = 0.89 + 0.45 × Ab.Ant. − 0.004 × Ab.Ant.^2^	0.43	17.2	0.00
Ab.Orth. = 0.11 + 0.19 × Ab.Col. − 0.01 × Ab.Col.^2^	0.16	4.2	0.02
Ab.Orth. = 0.10 + 0.21 × Ab.Tot.Pred. − 0.01Ab.Tot.Pred.^2^	0.19	5.3	0.01
Ab.Eux. = − 0.10 + 0.04 × Ab.Ant. − 0.01 × Ab.Ant.^2^	0.13	3.3	0.04
Ab.Lyr. = 7.62 + 0.19 × Ab.Aranei	0.23	13.8	0.00
Ab.Lyr. = − 0.12 + 0.17 × Ab.Ara	0.21	12.3	0.00
Ab.Lyr. = − 0.23 + 0.16 × Ab.Tot.Pred	0.21	12.2	0.00
Ab.Pol. = − 0.76 + 0.74 × Ab.Ant. − 0.01 × Ab.Ant.^2^	0.17	4.7	0.01
Ab.Pol. = − 3.30 + 0.71 × Ab.Tot.Pred. − 0.01 × Ab.Tot.Pred.^2^	0.22	6.3	0.00
Ab.Ts. = − 0.97 + 0.74 × Ab.Ant. − 0.01 × Ab.Ant.^2^	0.17	4.7	0.01
Ab.Ts. = − 3.42 + 0.70 × Ab.Tot.Pred. − 0.01 × Ab.Tot.Pred.^2^	0.22	6.2	0.00
Ab.Ts. = − 1.41 + 2.01 × Ab.Col. −0.08 × Ab.Col.^2^	0.19	5.1	0.01
Ab.Spid. = 1.54 + 0.26 × Ab.Pol	0.35	24.3	0.00
Ab.Spid. = 1.62 + 0.26 × Ab.Ts	0.33	22.4	0.00
Ab.Aranei = 0.28 + 0.28 × Ab.Ts	0.41	31.4	0.00
Ab.Tot.Pred. = 10.15 + 2.00 × Ab.Col	0.38	27.9	0.00
SR.Col = 2.00 + 0.40 × SR.Pol. − 1.71 × SR.Pol.^2^	0.30	9.6	0.00
SR.Col. = 0.93 + 1.15 × SR.Pred. − 0.18 × SR.Pred.^2^	0.27	8.4	0.00
SR.Pol. = 0.16 + 0.51 × SR.Hem. − 0.10 × SR.Hem.^2^	0.17	4.6	0.02
D.Orth. = 0.04 + 0.24 × D.Spid. − 0.02 × D.Spid.^2^	0.24	7.3	0.00
D.Orth. =—0.01 + 0.18 × D.Tot.Pred. − 0.01 × D.Tot.Pred.^2^	0.22	6.4	0.00
D.Ant. = 4.54 + 0.53 × D.Hem	0.10	5.3	0.03
D.Ant. = 3.72 + 1.21 × D.Spid. – 0.08 × D.Spid.^2^	0.23	6.7	0.00

ANOVA. n = 48, degrees of freedom: treatment = 1, replicates = 23, and of residue = 23.

### Predators and phytophagous insects

The abundance of Araneidae and spiders reduced (*P* < 0.05) the number of the leafminer *Lyriomyza* sp.; the abundance of protocooperating ants reduced phytophagous Coleoptera, *Euxesta* sp., pollinators, and *T. spinipes*; the abundance of total predators reduced phytophagous Orthoptera, pollinators and *T. spinipes*; and the abundance of *Polybia* sp. reduced Clytini. The diversity and species richness of total predators reduced (*P* < 0.05) the numbers of phytophagous Coleoptera and Orthoptera, respectively. On the other hand, the abundance of pollinators and *T. spinipes* increased (*P* < 0.05) spiders; the abundance of *T. spinipes* increased Araneidae; the abundance of phytophagous Coleoptera increased total predators and the leafminer *Lyriomyza* sp.; the abundance of Lepidoptera caterpillars and *T. spinipes* increased *M. religiosa*; the abundance of the leafminer of *Lyriomyza* sp. and *T. spinipes* increased *Podisus* sp.; the abundance of *Polybia* sp., spiders and protocooperating ants increased phytophagous Hemiptera; the abundance of Oxyopidae (Araneae) increased *Euxesta* sp.; the abundance of *Cratosomus* sp., phytophagous Hemiptera and Orthoptera increased Araneidae; the abundance of *Disonycha brasiliensis* Lima (Coleoptera: Chrysomelidae) and Tettigoniidae (Orthoptera) increased *Aphirape uncifera* Tullgren (Araneae: Salticidae). The diversity of Orthoptera increased (*P* < 0.05) the abundance of *A. uncifera*; the diversity of phytophagous Hemiptera increased protocooperating ants; the species richness of phytophagous Coleoptera and pollinators increased the abundance of *M. religiosa* and *Podisus* sp.; and the species richness of phytophagous Orthoptera and pollinators increased the abundance of Araneidae (Table 4, Fig. 1).

## Discussion

The highest ecological indices (abundance, diversity and species richness) of phytophagous, pollinator and predators arthropods on *T. argentea*, fertilized with dehydrated sewage sludge (DSS), are related to a higher nitrogen levels^6^ and consequently a better development of these plants (e.g. > leaves/tree =  > ETIB)^5,15^. The apparent competition between Coleoptera and Hemiptera for space and food, and the negative effect between protocooperating ants and phytophagous Coleoptera, are in accordance to findings on *Caryocar brasiliense* Camb. (Malpighiales: Caryocaraceae) trees^19,21^.

The highest ecological indices of phytophagous Cerambycidae, *Cerotoma* sp., *Cratosomus* sp., *Euxesta* sp., *Lamprosoma* sp., Lepidoptera caterpillars, *Parasyphraea* sp., *Psiloptera* sp., *T. collaris* and Hemiptera, ; pollinator *T. spinipes*; predators Araneidae, Salticidae, Pentatomidae and *Polybia* sp.; protocooperating ants and ecological processes (herbivory) on *T. argentea* trees fertilized with DSS, may be due to the highest numbers of leaves of this plant (> ETIB). Leaves are food resource with a better quality for these phytophagous insects, which in turn may attract a higher number of predators. Such an observation confirms the first hypothesis (i.e. ETIB), that the diversity and abundance of phytophagous insects, pollinators and their predators are usually higher on larger trees with higher leaf mass^5,15–17^. Thus, trees such as *T. argentea*, may seem as islands (as proposed by ETIB), and those with lower leaf mass present a higher chance to get extinct the endangered species^5,17,22,23^. In addition, the number of free amino acids and proteins in leaves, pollen and/or nectar production and quality (more protein and amino acids) in flowers, are superior in plants with higher nitrogen fertilization levels, e.g. *T. argentea* trees fertilized with DSS, increasing the attractiveness to phytophagous and pollinator insects^6,24–26^. Dehydrated sewage sludge used as a biofertilizer improved the macrofauna recovery, including scarab beetles’ larvae and adults in degraded soils of the Cerrado (Brazilian Savanna) biome^27^.

The abundance of phytophagous Hemiptera and pollinators (e.g. *T. spinipes*) reduced the number of phytophagous Coleoptera; whilst this insect order reduced the numbers of Orthoptera and *T. spinipes*, as well as those of phytophagous Hemiptera and phytophagous Orthoptera reduced Clytini. These correlations confirm the second hypothesis that there was competition between those insect groups for space and feeding. Moreover, protocooperating ants, associated with phytophagous Hemiptera, for instance, may have attacked beetles. However, further studies are needed to elucidate this hypothesis. Competition between defoliators (e.g. Coleoptera), sucking and galling insect species for space and feeding was observed on *C. brasiliense* trees^17,19^.

*Trigona spinipes*, by flying in flocks with aggressive behavior, chases other pollinators, such as *Apis mellifera* L. and *Tetragonisca angustula* Latreille (Hymenoptera: Apidae)^28^, and also likely other insects (e.g. beetles); beyond damages shoot and plant growth tissues to remove fibers for nests construction^5,29,30^. Food web studies are intricate due to interactions among host plants, phytophagous, predators and parasitoids insects, soil and climatic conditions^31^. Only a few studies have examined food webs in complex ecosystems, such as in the Cerrado^18,31,32^.

Spiders, the dominant predators group (excluding the protocooperating ants), correlated negatively with some phytophagous insects (e.g. *Lyriomyza* sp. and Orthoptera), confirming the third hypothesis on the negative correlation between phytophagous insects and predators. On the other hand, *T. spinipes* is perhaps the major prey to spiders on *T. argentea* trees. Spiders are important in the biological control of phytophagous (r = − 0.73; P = 0.00) and leafminer insects (r = − 0.62; P = 0.01) on *C. brasiliense* trees^19,21^. Spiders are important in pest control in agroforestry systems, especially in tropical regions^21,33–35^ since a wide range of pest insects can get caught in their webs, resulting in deaths^36^. The importance of these arthropods for biological control was confirmed by population reduction of *Epiphyas postvittana* (Walker) (Lepidoptera: Tortricidae) on *Malus domestica* Bork (Rosaceae) and *Phyllocnistis citrella* Stainton (Lepidoptera: Gracillariidae) on *Citrus sinensis* (L.) Osbeck (Rutaceae)^37,38^. In addition to spiders, the protocooperating ants were very abundant on *T. argentea* trees fertilized with DSS, probably due to the highest numbers of phytophagous insects—protocooperation^39–41^. The increased abundance of protocooperating ants reduced the numbers of phytophagous Coleoptera and *T. spinipes* on *T. argentea* trees, as observed in *C. brasiliense*, where the highest number of these ants reduced defoliation by beetles^19,21^. In addition, ants are bioindicators in the recovery of degraded area because they respond quickly to environmental complexity and by interacting mutually with other insects^42–45^. The abundance of the predatory wasp *Polybia* sp. was higher on fertilized plants probably due to a higher numbers of caterpillars (Lepidoptera) and the leafminer *Lyriomyza* sp.. Predatory wasps (Vespidae) are important natural enemies in agricultural systems such as *Brassica campestris* L. and kale *B. oleracea* L. var. *acephala* DC (Brassicales: Brassicaceae); Arabian coffee *Coffea arabica* L. (Gentianales: Rubiaceae) and tomato *Solanum lycopersicon* L. (Solanales: Solanaceae), preying mainly on caterpillars and leafminers (Lepidoptera)^46–49^.

In general, arthropod predators on *T. argentea* trees reduced herbivory by insects. However, in a few cases the presence of arthropod predators increased the numbers of phytophagous such as the leafminer *Lyriomyza* sp., likely by reducing competition with other more dominant groups (e.g. phytophagous Coleoptera). It shows how complex are interactions in food webs in natural and agroforestry systems^18,19,21,31,32,36^. Predators are often generalist in their feeding habits, and the greatest complexity of canopy architecture increases niches options for phytophagous insects and consequently for the natural enemy diversity^50^. For example, sewage sludge increases the richness of the ground beetle Carabidae (Coleoptera) in the area of Oxford, USA^51^.

The largest *T. argentea* tree canopy size (> ETIB) fertilized with DSS may explain the largest abundance of phytophagous insects (> defoliation), pollinators and predators, showing that this plant is adequate to recovery degraded areas. There was competition between groups of phytophagous insects and predator arthropods in high populations and consequent herbivory reduction.

## Material and methods

### Study

The study was conducted in a degraded area at the “Instituto de Ciências Agrárias (ICA)” of the “Universidade Federal de Minas Gerais (UFMG)”, Montes Claros, Minas Gerais, Brazil (S 16º51′38″ W 44º55′00″ 943 m) from March 2015 to February 2017 (24 months; arthropod collection period). The area presents soil loss and changes in soil chemistry and hydrology due to degradation^52,53^. Köppen’s climate classification^54^ defines this area as tropical dry climate; annual rainfall, 1,000–1,300 mm, with dry winter; annual mean temperature, ≥ 18 °C. The type of soil is litolic neosoil^55^ and chemical and physical details were described^5^.

### Study design

Seeds were collected from five-years old *Terminalia argentea* trees at ICA/UFMG campus before sowing. *Terminalia argentea* seedlings were produced in March 2014 by sowing one seed per plastic polybag (8 × 12 cm), and these kept in a nursery covered with black shed net. The mixed substrate contained 30% organic materials (i.e. two parts of debris gardening pruning < 5 cm in length, and one of brown bovine manure), 30% clay soil, 30% sand, and 10% of mineral fertilizer (i.e. 160 g reactive natural phosphate per seedling)^5^. The soil pH in the pits (40 × 40 × 40 cm) was corrected with dolomitic limestone with anhydrous carbonate mineral composed of calcium magnesium carbonate (90% relative total neutralization power) (187 g per pit), increasing base saturation to 50%^56^. Natural phosphate (80 g per pit), fritted trace elements (FTE) (10 g/pit), and marble roch dust (1 kg per pit) were added when needed. Thirty-centimeters tall *T. argentea* seedlings were planted in pits in a two-meters spacing , in six parallel lines on flat terrain with two-meters spacing lines, with four trees per treatment (fertilized or not with dehydrated sewage sludge—DSS) per line. The seedlings in experimental area were supplied with water until the beginning of the rainy season. The seedlings with five-cm long branches were pruned with a sterilized razor, eliminating the additional shoots (i.e. others different from the leader shoot) and branches up to 1/3 of crown height. The experimental design was in random blocks with two levels of fertilization (i.e. a single dose of 20 L of DSS per pit or none fertilization) and 24 replications with one plant each^5^.

DSS (with 5% mean moisture content) was obtained from a sewage treatment plant (STP) in Juramento, Minas Gerais, Brazil. The STP is operated by the Minas Gerais Sanitation Company – “Companhia de Saneamento de Minas Gerais S.A. (COPASA)”. The STP is highly efficient, removing more than 90% of the organic material from the domestic waste water. The sewage sludge is dumped off into coarse sand tanks, staying there for three months to reduce the amount of thermotolerant coliforms (and other pathogenic microrganisms) and reach the ideal levels for agricultural use that is < 10^3^ of the most likely number per g of total solids (as recommended by the National Council for the Environment—“Conselho Nacional do Meio Ambiente—CONAMA”). The chemical and biological characteristics of the DSS were described^5,6^.

### Arthropods

Insects and spiders were visually counted, every two weeks, on the adaxial and abaxial surfaces of the leaves between 7:00 and 11:00 AM at the apical, middle and basal canopy in the northerly, southerly, easterly and westerly directions, in 12 leaves per plant (i.e. 27,648 leaves from 48 *T. argentea* trees) during 24 months. Only insects and spiders collected for identification were removed from trees during the assessment. At least three specimens per insect or spider species were collected using aspirator, stored in glass flasks with 70% ethanol or mounted, separated into morphospecies, and sent for identification. Insect defoliation was assessed visually as the leaf area loss on a 0–100% scales with 5% increments for removed leaf area^57,58^.

### Ecological indices

To avoid pseudoreplication, mean numbers of data per tree were ever used. Ecological indices (abundance, diversity, and species richness) were calculated for each species per tree in the treatments (fertilization or not with DSS) using the software BioDiversity Professional, Version 2^59^. The arthropod diversity was calculated using the Hill’s formula^60,61^ and the species richness with the Simpson indices^62,63^. The predator (i.e. insects and spiders) and prey ratio on *T. argentea* was calculated per tree. Predators were classified as spiders (most important group), predators (predators + spiders) and total predators (spiders + predators + protocooperating ants).

### Statistical analyses

Data on defoliation percentage, abundance, diversity, and species richness of phytophagous insects, pollinators and predators were submitted to non-parametric statistical hypothesis, the Wilcoxon signed-rank test (*P* < 0.05)^64^, using the statistical program “Sistema para Análises Estatísticas e Genéticas” (SAEG), version 9.1^65^. Simple regression analyzes and parameters (*P* < 0.05) were performed with SAEG to test the interactions between groups of phytophagous, pollinators and predators, and foliar mass (see^41^).

The Spearman correlation matrix, among the most significant characteristics, was calculated. The matrices were submitted to correlation networks^66^. Edge thickness was controlled by application of a cut value of 0.28 (from which the Spearman correlation becomes significant, meaning that only edges with *|*r_ij_*|≥ *0.28 are highlighted). These analyses were performed in R version 3.4.1^67^. The correlation network procedure was performed using the package *qgraph*^66^.

### Ethics

No specific permits are required to *Terminalia argentea* tree in Brazil. The laboratory and field studies did not involve endangered or protected species.

## Data Availability

All data generated or analyzed during this study are included in this manuscript.
